# Cellular immunity induced by a recombinant adenovirus- human dendritic cell vaccine for melanoma

**DOI:** 10.1186/2051-1426-1-19

**Published:** 2013-11-18

**Authors:** Hadas Prag Naveh, Lazar Vujanovic, Lisa H Butterfield

**Affiliations:** 1 Department of Medicine, University of Pittsburgh Cancer Institute, 5117 Centre Avenue, Suite 1.27, Pittsburgh, PA 15213, USA; 2 Department of Surgery, University of Pittsburgh Cancer Institute, 5117 Centre Avenue, Suite 1.27, Pittsburgh, PA 15213, USA; 3 Department of Immunology, University of Pittsburgh Cancer Institute, 5117 Centre Avenue, Suite 1.27, Pittsburgh, PA 15213, USA; 4 University of Pittsburgh Cancer Institute, 5117 Centre Avenue, Suite 1.27, Pittsburgh, PA 15213, USA

**Keywords:** Adenovirus, Dendritic cells, T cells, Cytokines, Cancer vaccines

## Abstract

**Background:**

Human Adenoviral vectors (HAdV) are immunogenic vectors which have been tested in many vaccination and gene therapy settings. Dendritic cells (DC) transduced by genetically engineered HAdV-5 (HAdV-5/DC), are investigational cancer vaccines being tested clinically. We have previously examined immune responses to HAdV-5 -encoded melanoma tumor antigens. Here, we determined whether the HAdV-5/DC also present immunogenic HAdV-5 vector-derived antigens, and characterized the cellular immune response to the viral as well as encoded melanoma tumor antigens.

**Methods:**

Both CD4^+^ and CD8^+^ HAdV-5-specific T cell responses were examined *in vitro*, with cells from both 8 healthy donors (HD) and 2 melanoma patients. PBMC were stimulated weekly with HAdV-5/DC and responses were examined after each stimulation. We also tested HAdV-5 neutralizing antibody levels and natural killer (NK) cell and regulatory T cell (Treg) activation and expansion *in vitro*.

**Results:**

HAdV-5/DC rapidly induced a high frequency of type 1 cytokine producing HAdV-5-specific CD8^+^ and CD4^+^ T cells. IFNγ and TNFα-producing T cells predominate. Those with pre-existing cellular memory to HAdV-5 had more robust responses to the HAdV-5 as well as tumor-associated antigens. NK cells are activated while Treg are only minimally and transiently expanded.

**Conclusions:**

This study demonstrates that HAdV-5/DC promote strong type I cellular immunity to viral vector-derived antigens as well as to the encoded tumor antigens. The cytokine and chemokine milieu produced by HAdV-5/DC and the activated HAdV-5-specific T cells may enhance responses to encoded tumor antigens as well. These properties make HAdV-5/DC a cancer vaccine capable of activating type 1 virus and tumor antigen-specific immunity in a cooperative way.

## Background

Recombinant Human adenovirus serotype 5 (HAdV-5) is the most commonly used gene transfer vehicle with a long safety record [1]. The HAdV-5 36 kb genome (double stranded DNA) is well characterized, and used in many experimental and clinical settings [2]. Its systemic use in vaccination and gene therapy has been limited by pre-existing immunity and the presence of neutralizing antibodies in environmentally exposed individuals. Neutralizing antibodies, directed mainly against capsid hexon loops, are serotype specific. Approximately 45% of adults in the United States possess neutralizing antibodies to HAdV-5. A variety of other Adenoviral subtypes and deletion mutants have been investigated (Ad28, Ad35 and “gutless” Adenovirus), in an attempt to circumvent pre-existing immunity in exposed individuals.

We have utilized E1/E3-deleted, replication deficient tumor antigen-encoding HAdV-5 *in vitro* and *in vivo*, to promote tumor antigen-specific immunity (reviewed in [3]. As previously shown, dendritic cells (DC) transduced with an HAdV-5 (HAdV-5/DC) encoding full length MART-1 (HAdV-5 MART1), activate MART-1 specific CD8^+^ and CD4^+^ T cells *in vitro*[4,5] and *in vivo*[6]. Similarly, DC transduced with an HAdV-5 encoding alpha fetoprotein (AFP, HAdV-5hAFP) activate CD8^+^ and CD4^+^ T cells to AFP *in vitro*, and are more efficient at T cell stimulation than AFP protein-fed DC [7-9]. Immunogenicity of HAdV-5/DC can be attributed to the fact that HAdV-5 transduction promotes a more mature DC surface phenotype, associated with a unique cytokine and chemokine secretion profile [10,11]. Additionally, transduction with HAdV-5 modulates components of MHC class I antigen processing machinery in DC [11]. Recently we showed that HAdV-5/DC can also promote NK cell chemotaxis *in vitro* and *in* vivo via IL-8/CXCL8 and IP-10/CXCL10 secretion and induce activation of both major subsets of NK cells, which requires cell-cell contact via cell surface TNF and IL-15 [12,13].

We have examined the cross-talk between HAdV-5 and DC, as well as downstream effects on transgene-specific T cell subsets and other lymphocytes. In addition to introducing a transgene, HAdV-5 engineering of DC also leads to expression of the remaining HAdV-5 genes encoded within the vector (derived from 28 genes and overlapping open reading frames) in the DC, and processing and presentation of viral coat proteins, such as the HAdV-5 hexon and fiber, released during HAdV-5 uptake in endosomes. The immunologic impact of the presentation of these viral proteins by transduced DC to T cells is yet unknown. Our previous preclinical murine models indicated that pre-immunization with HAdV-5 did not impact the antitumor immunity from an AdVMART-1-transduced DC vaccine [14], but detailed viral immunity assessments were not performed because mice are not permissive for HAdV infections.

Most previous studies examining humoral and cellular immunity to HAdV-5 have focused on directly injected vectors, and vectors involving HIV antigens encoded by HAdV-5. Humoral responses to HAdV-5 were shown to be critical to the efficacy of an HIV vaccine [15]. It has been shown that HAdV-5 neutralizing antibody levels were unrelated to T cell responses to hexon or E2A viral proteins, and that the levels of HAdV-5-specific CD4^+^ T cell responses varied with the specific deletions in the HAdV-5 backbone [16]. Multiplex cytokine profiling showed that a broad Th1/Th2/regulatory profile resulted from MRKAd5 HIV gag immunization of healthy volunteers [17].

Other previous clinical studies utilizing recombinant HAdV-5 vectors encoding additional foreign viral antigens (HIV, EBV, CMV) have examined some aspects of immune response to the HAdV-5 viral antigens [14,15,18-23]. Other reports in the literature utilize replication-competent HAdV-5 which still encode the E1a/E1b transactivators (including oncolytic viruses), leading to a high level of viral gene transcription and translation, and often lytic growth in infected human cells. However, the responses to recombinant HAdV-5 which encode normal, non-mutated self-antigens, like melanoma lineage tumor antigens, may have a unique profile due to the colocalized presentation of both classes of antigens (self and viral). Such self-antigen encoding HAdV-5 have been tested in transduced DC clinical trials [6,22,23], but the immunity to the HAdV-5 aspects of these vaccines has not been examined to date.

We have recently developed a new melanoma vaccine clinical trial testing immunization with three full length melanoma tumor antigens (HAdV-5 TMM2, encoding Tyrosinase, MART-1 and MAGE-A6), instead of a single antigen [24], as in our previous trial [6]. While we have previously characterized the T cell responses to the encoded tumor antigens, we have not examined whether HAdV-5-specific cellular immune responses are also activated. Based on previous studies showing that the HAdV-5 capsid protein hexon encodes CD8 and CD4 T-cell epitopes [25,26], we hypothesized that HAdV-5-specific memory T cells may be detected in the periphery of environmentally exposed individuals, and that these responses might be quickly reactivated with HAdV-5/DC stimulation. We also hypothesized that these responses would be predominantly type 1, which might serve to skew the cellular environment in which the virally encoded tumor antigens are presented. Here, we have carefully examined the *in vitro* CD8^+^ and CD4^+^ T cell response to HAdV-5-specific antigens on DC transduced with a replication-deficient HAdV-5 (HAdV-5 TMM2). We find that a high frequency of type 1 CD8^+^ and CD4^+^ T cells are activated to the viral antigens and the overall cytokine milieu is type 1. We also find that NK cells in culture are activated, and regulatory T cells (Treg) remain at a low frequency *in vitro*, while activated CD4^+^ T cells expand. Importantly, HD and melanoma patients with pre-existing cellular HAdV-5-specific memory T cells showed potent HAdV-5 and melanoma antigen responses *in vitro* which were unrelated to humoral memory.

## Results and discussion

### HAdV-5/DC rapidly induce high frequencies of HAdV-5-specific CD4^+^ T cells

**Figure 1:**
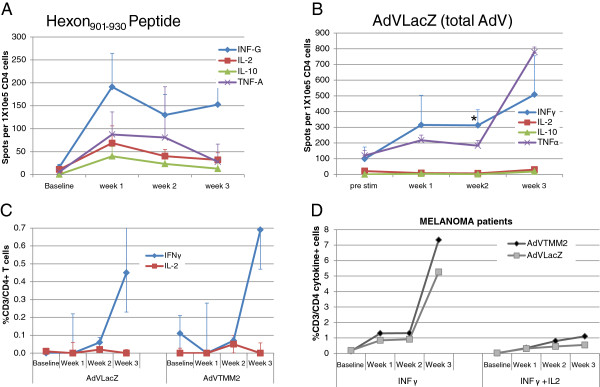
**HAdV-5-specific CD4**^**+ **^**T cell responses.** PBMC were cultured with autologous DC transduced with HAdV-5 TMM2 (baseline) for 1 week, followed by 2 restimulations with HAdV-5 TMM2/DC (weeks 1 and 2) and harvest (week 3). **(A and B)**: CD4^+^ T cells were purified and tested by direct ELISPOT for recognition of hexon_901-930_ peptide **(A)** or full length HAdV-5 (HAdV-5 LacZ, **B**). The mean frequency of cytokine producing cells from seven donors is shown. These cells were also tested for IFNγ and IL-2 production by flow cytometry, which was found to be low, averaging at 0.05% at week 3 (data not shown). **(C)**. Data from 7 independent HD is shown. Identically cultured melanoma patient cells responding to HAdV-5 LacZ or HAdV-5 TMM2 (HAdV-5 plus melanoma antigens) is shown (n = 2, averaged), for total IFNγ-producing cells and polyfunctional, simultaneous IFNγ + IL-2-producing cells **(D)**. Error bars represent standard error of the mean. *Significant response vs. baseline by single tailed student’s t-test (p < 0.05).

To define the CD4^+^ T cell response to HAdV-5 in HD, we stimulated PBMC with HAdV-5 TMM2-transduced DC (HAdV-5 TMM2/DC) *in vitro*, for a total of three weekly stimulations. This was chosen to mimic the three total vaccinations used in our current and previous clinical trials [6,27,28]. We hypothesized that we would expand high frequencies of type 1 T cells, based in part on the HAdV-5 virus-specific T cell adoptive transfer studies [20] which have demonstrated that HAdV-5-specific CD8^+^ and CD4^+^ T cells can have a high proliferative capacity, secrete IFNγ and show cytotoxicity towards HAdV-5-infected targets [21]. T cells were tested weekly, after each stimulation. We utilized two standardized, functional assays to measure responses, using the cytokine ELISPOT from purified T cell subsets, and flow cytometry with surface and intracellular cytokine staining. Mean HAdV-5-specific CD4^+^ T cell frequencies for 7 HD are shown in Figure 1A, 1B, and 1C.

While several promiscuous MHC class II epitopes have been described (Hex_571-600_, Hex_856-885_, Hex_901-930_) [29], we primarily detected responses to the HAdV-5 hexon_901-930_ (Hex_901-930_) epitope. As shown in Figure 1A, after only a single HAdV-5 TMM2/DC stimulation, a high frequency of Hex_901-930_-specific CD4^+^ T cells were expanded, producing primarily IFNγ, as well as TNFα and IL-2. Low frequencies of IL-10 producing cells were also detected from HD. TNFα or IL-10 responses to the Hex_571_ and Hex_856_ epitopes were not detected and there were few examples of IFNγ or IL-2-producing T cells detected specific to Hex_856_ (not shown). Since the HAdV-5 hexon capsid protein is very immunogenic and has been found to account for the majority of anti-HAdV-5 T cell reactivity in other studies [21], we hypothesized that other HAdV-5-derived epitopes might play an important role in HAdV-5/DC induced T cell responses. We used an E1/E3-deleted HAdV-5 encoding β-galactosidase (HAdV-5 LacZ), which has the same HAdV-5 backbone as HAdV-5 TMM2 and which also encodes the same 28 overlapping HAdV-5 gene products as HAdV-5 TMM2, to measure the total HAdV-5-specific T cell responses. While the hexon-specific responses peaked at one week (all measured cytokines), the total HAdV-5 response, largely consisting of IFNγ and TNFα-producing T cells, continued to increase through three weekly stimulations (Figure 1B). The difference in expansion kinetics between hexon-specific and total HAdV-5-specific CD4^+^ T cells may be due to the minimal levels of viral gene transcription and the presence of intact hexon protein in the coat of AdVTMM2. Comparing the CD4^+^ T cell response to the HAdV-5 backbone (HAdV-5 LacZ) with the response to both HAdV-5 and the three melanoma-associated antigens (MAA, HAdV-5 TMM2), a minimal IL-2 response was detected at week 2 and a stronger IFNγ response was observed after three stimulations, with specificity for both viral and melanoma antigens (Figure 1C). The frequency of CD4^+^ T cells which were polyfunctional (producing IFNγ and IL-2 simultaneously by flow cytometry) was low, averaging 0.05% of CD4^+^ T cells at the peak at week 3 (not shown).

Cancer patients can be immune suppressed, due to multiple immune modulatory effects of both tumor cells and many standard of care cancer treatments. Therefore, it was important to include cancer patient cells in the analysis. We used IFNγ-producing HAdV-5-specific CD4^+^ T cells obtained from two different melanoma patients also expanded to high frequencies *in vitro*. Despite these virus-specific T cell frequencies, the T cells specific to the three encoded melanoma antigens were still detectable, at over 1% frequency, at week 3 (Figure 1D). Interestingly, the frequency of polyfunctional CD4^+^ T cells was higher in both of the patient cell cultures (0.3 and 0.8% at week 3, not shown) than in HD cultures, indicating that the tumor and therapeutic treatments received did not inhibit HAdV-5 or tumor antigen responses. Conceivably, immunotherapies received by these patients may have supported previous expansion of the melanoma antigen-specific T cells, allowing for memory responses.

### HAdV-5-specific CD8^+^ T cell responses are rapidly induced by HAdV-5/DC

**Figure 2:**
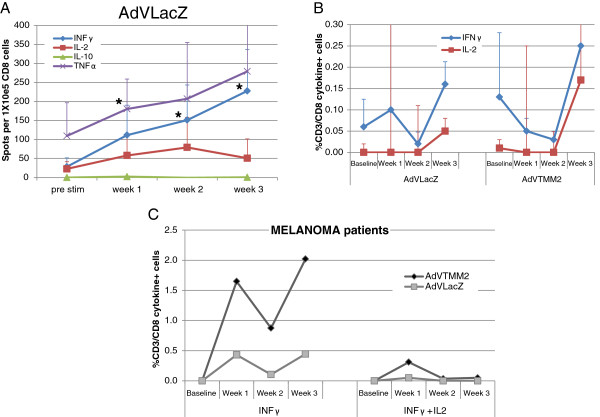
**HAdV-5-specific CD8**^**+ **^**T cell responses.** CD8^+^ T cells were cultured as described in Figure 1, and were tested for responses to HAdV-5 and melanoma antigens. **(A)** The mean frequency of CD8^+^ T cells from 7 donors responding to HAdV-5 by ELISPOT is shown. **(B)** These cells were also tested for IFNγ and IL-2 production by flow cytometry. Data from 7 independent HD is shown. Identically cultured melanoma patient cells responding to HAdV-5 LacZ or HAdV-5 TMM2 (HAdV-5 plus melanoma antigens) is shown (n = 2, averaged), for total IFNγ-producing cells and polyfunctional, simultaneous IFNγ + IL-2-producing cells **(C)**. Responses were highest at week 3 for 5 of 7 donors and 1 of 2 patients. *Significant response vs. baseline by single tailed student’s t-test (p < 0.05).

To define the CD8^+^ T cells response to HAdV-5 in HD, we utilized the same culture and assay strategy. We tested the HLA-A2-restricted hexon_711-721_ peptide previously identified as the immunodominant hexon epitope, but did not reliably detect responses from any of our HLA-A2^+^ donors (data not shown). We did detect high frequency type 1 responses to the total HAdV-5 proteins (HAdV-5 LacZ) as shown in Figure 2. Cells producing TNFα and IFNγ predominated and showed greater frequencies with every subsequent stimulation, with IL-2 producing cells also detected (Figure 2A). Comparing the HAdV-5 and HAdV-5 + MAA responses, CD8^+^ T cells producing IFNγ and IL-2 (at a lower frequency) could be detected to both after 3 stimulations (Figure 2B). Comparing CD4^+^ and CD8^+^ T cell responses, there were more IFNγ-producing CD4^+^ T cells and more IL-2-producing CD8^+^ T cells expanded from the HD tested (Figures 1C and 2B). The frequency of polyfunctional HAdV-5-specific CD8^+^ T cells was also low, averaging 0.04% of CD8^+^ T cells at the peak at week two (not shown).

To confirm that the patterns observed in HD were representative of the responses in melanoma patients, we repeated the experiments with PBMC from the same HAdV-5anced stage melanoma patients (Figure 2C). While HAdV-5-specific cells were clearly expanded, the relative frequency of melanoma antigen to HAdV-5 viral antigen responses was much greater than in CD4^+^ T cells (Figure 1C). The frequencies of polyfunctional CD8^+^ T cells in the two patients were similar to that of the HD (0.05%, peak at week 1, not shown).

### Impact of HAdV-5-specific cellular memory responses

Because most individuals have been previously exposed to HAdV-5 in the environment, we expected that donors and patients would have variable but detectable levels of pre-existing cellular and humoral HAdV-5 immunity. Additional file 1: Figure S1 shows the range of anti-HAdV-5 neutralizing antibodies detected in our pool of donors and patients. These data show that the majority of donors and patients have been exposed to HAdV-5 and have detectable titers of neutralizing antibodies, as expected. The two melanoma patient’s sera did not differ appreciably from the HD sera tested. Importantly, the baseline titer of anti-HAdV-5 neutralizing antibodies did not correlate with the induced cellular response to HAdV-5 *in vitro*. This is similar to the observation of Koup et al. [16] in their study of intramuscular HAdV-5-HIV gag immunization.

**Figure 3:**
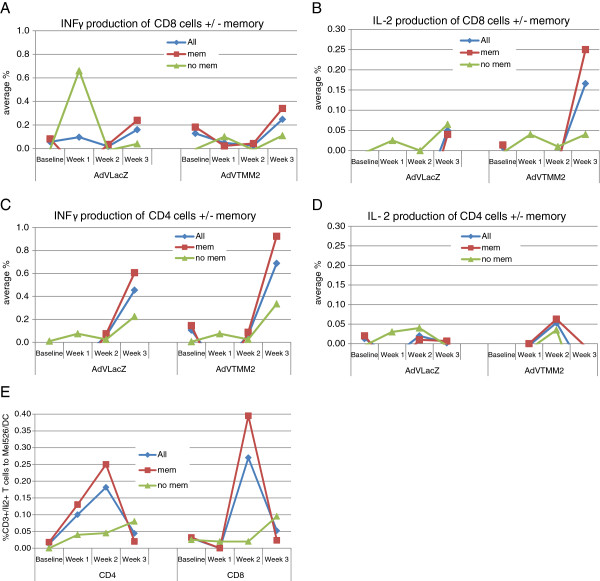
**Impact of baseline memory on induced responses.** The frequency of CD8^+^**(A and B)** and CD4^+^**(C and D)** T cells producing IFNγ **(A and C)** or IL-2 **(B and D)** to HAdV-5 (HAdV-5 LacZ) or HAdV-5 + melanoma antigens (HAdV-5 TMM2) are shown (“All”). Those HD with ≥ 0.03%^+^ baseline IFNγ and/or IL-2 responses were also analyzed separately, as having cellular memory responses (“mem”, n = 5) vs. those with < 0.03%^+^ cells, or no baseline cellular memory (“no mem”, n = 2). The frequency of CD8^+^ or CD4^+^ T cells producing IL-2 to DC loaded with Mel526 lysate are shown **(E)**.

We next determined the impact of baseline cellular immunity to HAdV-5 on the resultant expansion of T cells from HAdV-5/DC stimulation. By intracellular cytokine staining, all tested donors had at least 0.02% positive IFNγ or IL-2 producing CD4^+^ and/or CD8^+^ T cells, and the assay sensitivity is reported to be 0.01% or less [30]. To investigate the impact of baseline HAdV-5-specific cellular memory on the level of induced T cell responses to HAdV-5 antigens as well as MAA, we used a cut off of 0.03% baseline CD8^+^ or CD4^+^ positivity (IFNγ or IL-2) to compare the induced T cell responses between those with ≤0.02% positive HAdV-5-specific T cells (the lowest detected), to those with ≥ 0.03% positive HAdV-5-specific T cells. As shown in Figure 3, those with cellular memory responses (red symbols) generally induced higher frequency CD8^+^ IFNγ and IL-2 responses to HAdV-5 antigens and MAA after the three stimulations (Figure 3A-B). This baseline memory group also showed higher CD4^+^ IFNγ responses at 2–3 weeks to the HAdV-5 and HAdV-5 TMM2 (HAdV-5 + MAA) groups. The melanoma antigen-specific responses were stronger (with both IFNγ and IL-2) from those with baseline HAdV-5 cellular memory (Figure 3A-D). Importantly, the IL-2 producing T cells recognizing autologous DC pulsed with lysate from the melanoma cell line Mel526 (expressing Tyrosinase, MART-1, MAGE-A3/6 antigens, not shown) were also most frequently recognized by those with HAdV-5 cellular memory (Figure 3E). These data suggest that the higher frequency of HAdV-5-specific cells producing type 1 cytokines provides a supportive milieu for the MAA-specific T cells also being stimulated by the HAdV-5 TMM2/DC.

Our observation of robust expansion of type 1 cytokine producing HAdV-5-specific T cells is in sharp contrast to the lack of expansion of HAdV-5-specific T cells *in vivo* after HAdV-5-HIV gag vaccination [16]. This may relate in part to the difference in HAdV-5 backbone. The HIV gag-encoding vector in that study was not only E1 and E3 deleted, but also E4 deleted.

### HAdV-5 TMM2/DC promote NK cell activation *in vitro*

Our previous data showed that HAdV-5 LacZ/DC can activate NK cells [12]. Therefore, we examined the activation status of NK cell subsets in the HAdV-5 TMM2/DC cultures. The NK cells were separately analyzed as two major and three minor subsets (Additional file 2: Figure S2a), as described previously [31]. We observed that the frequency of total activated CD69^+^ NK cells (Additional file 2: Figure S2b) and activated IFNγ-producing NK cells (Additional file 2: Figure S2c) increased with HAdV-5 TMM2/DC stimulation. These data confirm that HAdV-5 TMM2/DC promote NK cell activation, and that changing the encoded transgene from xenoantigens β-galactosidase or luciferase to melanoma tumor antigens tyrosinase, MART-1 and MAGE-A6 does not change the ability to activate NK cells.

### HAdV-5 TMM2/DC do not induce regulatory T cell responses

**Figure 4:**
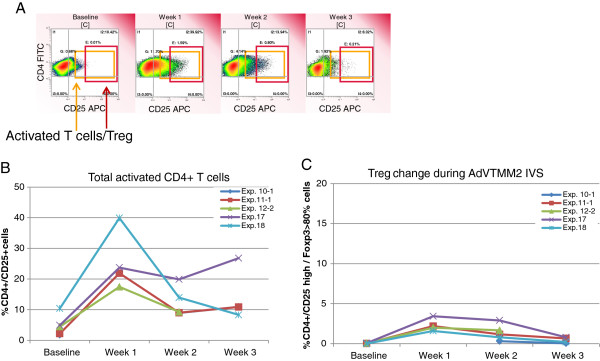
**Total activated CD4**^**+ **^**T cells and Treg expanded *in vitro*.** The cultured PBMC were tested weekly (without further stimulation) for the frequency of total activated CD4^+^ T cells (CD25^+^), and the subset which represent Treg (CD25^hi+^ and > 80% intracellular FoxP3^+^). The gating strategy for each population is shown **(A)**, total activated CD4^+^ T cells are in **(B)** and Treg shown in **(C)**.

There have been reports of certain types of DC activating suppressive Treg responses [32]. Such expansion of Treg would be an unwanted side effect of HAdV-5/DC stimulation in a cancer immunotherapy setting. Therefore, we determined the overall levels of both CD4^+^ T cell activation (regardless of antigen specificity), as well as levels of Treg in the HAdV-5/DC cultures by flow cytometry. As shown in Figure 4A and B, the frequency of activated (CD25^+^) CD4^+^ T cells were sharply increased after a single stimulation with HAdV-5/DC to 15-40% of the total CD4^+^ T cells, later decreasing to 8-27%. In contrast, the frequency of CD25^hi^ FoxP3^+^ CD4^+^ Treg detected was extremely low (<4%), and decreased to <1% after the third HAdV-5/DC stimulation (Figure 4C). This could be attributed to the fact that HAdV-5/DC have a unique level of maturation where they secrete low amounts of immunoregulatory cytokines (IL-10 and IL-13), and more immunostimulatory cytokines (TNFα, IL-15) [11].

### Cytokine and chemokine milieu

**Figure 5:**
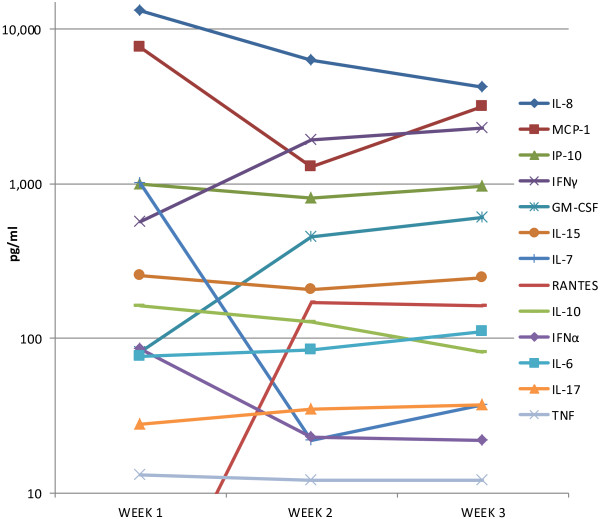
**Analytes in weekly HAdV-5 TMM2/DC cultures by Luminex.** Cell-free supernatents were collected after each 7 day culture period and frozen until simultaneous testing by multiplex Luminex 30-plex assay. The average levels for each analyte detected are shown at each time point, pooling 4–7 HD and 2 melanoma patients’ culture data.

As shown in Figures 1 and 2, we detected high frequencies of HAdV-5-specific T cells producing primarily IFNγ and TNFα. We also measured CD69 on all T cells, to determine the activation status on a population basis. At baseline, 2% of CD4^+^ T cells and 4% of CD8^+^ T cells were CD69^+^, and PMA/ionomycin activation resulted in 45-55% CD69 positivity (data not shown). The majority (up to 89%) of IFNγ and/or IL-2-producing cells were CD69^+^, as expected. Of the 11-24% which expressed CD69 following HAdV-5 LacZ/DC exposure, less than 1% generally produced IFNγ or IL-2 (Figures 1 and 2). Therefore, we hypothesized that additional cytokines were likely being produced by these CD69^+^ activated T cells. To more broadly characterize the cytokine milieu (created by both HAdV-5/DC and lymphocytes), we tested many soluble factors present in the HAdV-5/DC cultures at each weekly stimulation by multiplex Luminex assay. As shown in Figure 5, many cytokines and chemokines were detected. In addition to IFNγ and TNFα, MCP-1/CCL2, RANTES/CCL5, IL-7 and GM-CSF were highly produced. IL-7 has anti-apoptotic properties and may also serve to support T cell growth and GM-CSF may aid DC viability [33,34]. Based on our recent findings on NK cell crosstalk, we examined both CXCL8/IL-8 and CXCL10/IP-10, which we demonstrated are secreted by HAdV-5/DC and responsible for NK cell chemotaxis [13]. Both were detected at high levels, which would support NK cell chemotaxis at each weekly stimulation (as well as possibly chemotaxis of neutrophils and type 1 T cells, respectively). IL-15, which we showed is crucial for HAdV-5/DC-mediated NK cell activation (when trans-presented on the DC surface), and which has also been shown to support memory T cell homeostasis was also detected [35].

There have been reports of type 1 interferons (IFN) produced by a variety of human cells upon HAdV-5 exposure, which might be expected as part of an antiviral response [36]. HAdV-5 induction of IFNα has been shown to have a variety of positive and negative effects in the setting of recombinant HAdV-5 vaccines [37]. IFNα is also known to help mature DC, promote CD8^+^ T cell growth, support memory responses, and support NK cells [38]. In these *in vitro* cultures, we detected low levels of IFNα, generally under 100 pg/mL. These levels (which are well below physiologically achieved levels in high dose IFNα-treated patients) may be too low to promote DC maturation [10].

We did not detect VEGF or IL-2 by Luminex. As shown in Figures 1 and 2, IL-2-producing T cells were detected by intracellular cytokine staining, but levels of soluble IL-2, a potent T cell growth factor, were either below detection or more likely taken up by other cells in culture. In contrast, soluble IFNγ and TNFα, also produced by antigen-specific T cells, were within detection limits in the cultures. VEGF is a potent inducer of angiogenesis and skews immunity to support tumor growth, hence the lack of VEGF is a positive aspect of the HAdV-5/DC cross talk with lymphocytes. Lastly, we detected low levels of IL-17, which may be produced by pro- or anti-tumor T cells [39]. The role of Th17 cells in our system is not yet known, but these preclinical data suggest that these cells should be tested in our clinical trial patients.

When comparing HD to melanoma patient (results not shown), the patients had lower levels of IL-10, IL-6, IL-15, MCP-1, IL-Ira, IP-10, MIG and IL-8 and higher levels of MIP-1α and GM-CSF, although higher numbers of HD and patients are needed to determine the significance of these observations. We did not detect a correlation between neutralizing antibody levels and cytokines produced in HD or patient cell cultures.

A study of HD vaccinated intramuscularly with MRKAd5-HIV-1 gag tested the cytokine profiles of PBMC at 30 weeks post injection [17]. The array of analytes tested was similar to ours. IL-8, MCP-1 and IP-10 were found in similar concentrations and were also the highest produced analytes in their study, supporting the potential *in vivo* relevance of our findings. However, there were also several differences between their findings and ours, including higher levels of IFNγ, GM-CSF, lower levels of IL-6 and IL-10 and no VEGF detected in our study. It will be important to confirm the quality and frequency of these cellular and secreted molecule responses presented with the *in vivo* responses in patients vaccinated with HAdV-5 TMM2/DC.

## Conclusions

We find that DC engineered with a replication-deficient Ad5 vector encoding three melanoma antigens activate high frequencies of HAdV-5-specific CD4^+^ and CD8^+^ T cells. These T cells produce both IFNγ and TNFα effector cytokines, which create a type 1 cytokine milieu, and which have recently been shown to be important to promote senescence in tumors [40]. These cellular responses were independent of the level of anti-HAdV-5 humoral immunity. Interestingly, those with pre-existing cellular memory to HAdV-5 had superior responses not only to the HAdV-5 but also to the melanoma antigens. This suggests that the type 1 virus-specific T cell response may support a response to the vector-encoded melanoma antigens. We will investigate this in our ongoing HAdV-5 TMM2/DC vaccine clinical trial, *in vivo*. Additionally, we reaffirmed the ability of HAdV-5/DC to activate NK cells and not Treg, which can further aid in the creation of the type 1-skewing milieu, as well as provide an innate cytotoxic response against tumors.

## Methods

### Cell culture and cell lines

Mel526 is a human melanoma cell line which expresses MART-1/Melan-A, tyrosinase and MAGE-A3/6. The cell lines (Mel526, HEK293, A549) were cultured in Advanced RPMI 1640 medium supplemented with 10% fetal bovine serum (FBS), 1% penicillin streptomycin and 1% L-glutamine (all reagents from Invitrogen), in a humidified 37°C incubator under 5% CO_2_ tension. Mel526 lysate was prepared by repeated freeze/thaw of a cell pellet.

### Peptides

Peptides were synthesized at the University of Pittsburgh Peptide Synthesis Facility using standard f-moc technology. Stock solutions were prepared in DMSO (10 mg/mL) and were kept at -80°C until use. HAdV-5 hexon_711–721_ TFYLNHTFKK and the HAdV-5 promiscuous hexon peptides: _571–600_ NLLLLPGSYTYEWNFRKDVNMVLQSSLGND, _856–885_ VDSITQKKFLCDRTLWRIPFSSNFMSMGAL and _901–930_ LDMTFEVDPMDEPTLLYVLFEVFDVVRVHR.

### Virus amplification, purification and titer

Virally infected HEK 293 cells (ATCC, #CRL-1573) were freeze/thawed and the cleared supernatant was layered onto a CsCl step gradient (1.2 g/mL/1.4 g/mL) and centrifuged at 35,000 rpm 1.5 h. The virus band was dialyzed (Pierce Slide-a-lyzer 10 K, Tris/MgCl2/glycerol), and concentrated (Millipore) in formulation buffer (2.5% glycerol (w/v), 25 mM NaCl and 20 mM Tris–HCl, pH 8.0; Clontech), aliquoted and titered (Adeno-X Rapid Titer Kit, Clontech). HAdV-5 LacZ was obtained from the University of Pittsburgh Vector Core. HAdV-5 TMM2 encodes a CMV-Tyrosinase-IRES-MART-1-SV40pA cassette in the E1 region, and an RSV-MAGE-A6-SV40pA cassette in the E3 region [24].

### RT-PCR

Total RNA was isolated (Qiagen Kit; Qiagen) and quantified by UV spectrophotometry. RT-PCR was performed using Random Primers, and SuperscriptTM III Reverse Transcriptase, Ampli Taq Gold polymerase. Sequenced-specific primers were as follows:

Tyrosinase, Forward: 5’-ATT-CCA-TAT-TGG-GAC-TGGCGG-GAT-3’/Tyrosinase

Reverse: 5’-CAA-TGG-GTG-CAT-TGG-CTT-CTG-GAT-3’

MART-1, Forward: 5’ – TGC-AGA-TAT-CCA-TCA-CACTGG-3’

Reverse: 5’ – GGA-GGG-GCA-AAC-AAC-AGA T – 3’

MAGE-A6, Forward: 5’- AGT-AGG-AAG-GTG-GCC-AAGTTG-GTT-3’

Reverse: 5’- TAT-TGG-GTG-AGC-TTC-TTGGGA-3’

β-Actin, Forward: 5’-GGC-ATC-GTG-ATG-GAC-TCC-G-3’

Reverse: 5’-GCT-GGA-AGG-TGG-ACA-GCG-A-3’

All primers were prepared by Integrated DNA Technologies, Inc. PCR reactions for β-Actin, Tyrosinase and MART-1 were 32 cycles (61.7°C annealing), and for MAGE-A6, 30 cycles (55°C annealing) were used.

### Isolation of peripheral blood mononuclear cells (PBMC)

Peripheral blood was obtained from 8 different HLA-A2^+/-^/HLA-DR4^+/-^ normal donors with their written consent, under an IRB-approved protocol (UPCI 04–001, L. H. Butterfield, PI).

Melanoma patient PBMC were obtained under Protocol UPCI 96–099 (J. M. Kirkwood, PI) at a time point distant from any treatment. Patient #1 was diagnosed at stage 1, and 5 years post diagnosis, an inguinal metastases was found and she was treated with lymphadenectomy + high dose Interferon alpha-2b. Patient #2 was diagnosed at stage 4 and received several previous treatments: Temozolomide, Interferon, high dose IL-2, BCG plus tumor vaccine. PBMC were separated from blood using Ficoll-Hypaque gradient centrifugation (Cellgro; Mediatech, Inc.) with average viability of 98%. Additional buffy coats from healthy donors were obtained from the Central Blood Bank (Pittsburgh, PA).

### Generation of dendritic cells (DC) and DC culture

DC were prepared as described [24] with some modifications. Mononuclear cells (7–8 x 10^7^) were plated in T-75 flasks (Costar) in RPMI 1640/PSF/5–10% human AB serum for 2 h at 37°C in a humidified CO_2_ incubator. The non-adherent cells were removed by gentle rinsing with PBS, and the loosely-adherent cells were cultured in medium with 800 U/mL GM-CSF (Sargramostim; Amgen) and 500 U/mL IL-4 (Schering Plough) for 6–7 d. The DC were harvested by TrypLE select, and had average 90% viability at harvest, and 85% viability post HAdV-5 transduction. DC were either transduced at moi = 500 pfu: DC or loaded with Mel526 lysate at 10 μg/ml for 2 hours at 37°C in serum-free medium, then washed.

### *In vitro* stimulation

PBMC from HD or patients were stimulated *in vitro* using HAdV-5/TMM2 transduced DC in a ratio of 1:10–1:40 (DC:PBMC). 25 ng/ml of IL-7 was added once at setup of the IVS. 30U/ml IL-2 was added on day 3 of each IVS to maintain viability. Stimulated PBMC were collected weekly, washed, and re-stimulated with freshly transduced DC. A portion of PBMCs were collected from culture weekly and cryopreserved.

### Selection of CD8^+^ and CD4^+^ lymphocytes

Before ELISPOT assays, the CD8^+^ and CD4^+^ T cells were sequentially isolated by positive and negative MACS, respectively, with the use of immunomagnetic beads following the manufacturer’s instructions (Miltenyi Biotech). Resulting cell populations were 95% CD8^+^-positive according to FACS analysis. Viability at culture harvest for analysis averaged 82%.

### ELISPOT assays

Multi-screen HA plates (Millipore, MAHAS4510) were coated with 4–10 ug/mL of monoclonal capture Ab anti-human IFNγ (1-D1K), IL-2 (IL-2-1), IL-10 (9D7) or TNFα (TNF3/4; all from MabTech), in PBS overnight at 4°C. After blocking the plates with RPMI/10% AB (1 h, 4°C), CD8^+^ or CD4^+^ T cells were plated at 10^5^ cells/well in duplicate or triplicate wells. Autologous DC (0.5–1 x 10^5^ well) were pulsed with different peptides or transduced with HAdV-5, rinsed and plated. Control wells contained T cells (alone or with 0.2 ng/ml PMA + 0.2 μM ionomycin), T cells with unloaded DC (“no peptide”) or negative antigen (HIV gag or alpha fetoprotein peptides tested in a subset of ELISPOT assays) pulsed DC. Cells were removed and captured cytokine was detected by corresponding biotinylated mAb (MabTech) at 2 μg/mL in PBS/0.5% BSA. After washing, Avidin Peroxidase Complex (Vectastain Elite Kit) was added for 60 min. After rinsing, peroxidase staining was performed with 3-amino-9-ethyl-carbazole (AEC, Sigma) and stopped by rinsing the plates under tap water. Spot numbers were automatically determined with ImmunoSpot imaging system from Cellular Technology, Ltd. Spots detected to “medium only” or T cells only were < 10. Spots detected to PMA + ionomycin were > 1,000 on average. To calculate the number of responding T cells, the mean number of spots detected with DC alone were subtracted from mean spot numbers induced by antigen-loaded DC. The range of DC only background subtracted varied by cytokine examined.

### Neutralizing antibody assay

Anti-HAdV-5 neutralizing antibodies were detected using serial dilutions of serum (1:4 to 1:512) cultured with an indicator cell line, A549 (ATCC), followed by transduction with an HAdV-5-encoding enhanced Green Fluorescent Protein (HAdV-5 eGFP), as described [36]. The cells were tested for the MFI of eGFP by flow cytometry.

### Luminex assay

Cell-free supernatants were collected from cultures and frozen at -80°C. They were subsequently thawed and simultaneously analyzed with the multiplex Luminex assay (Invitrogen) per manufacturer’s protocol in a BioRad reader (UPCI Immunologic Monitoring Laboratory). The following analytes were tested: GM-CSF, IFNγ, IP-10, MCP-1, TNFα, IL-1β, IL-2, IL-4, IL-5, IL-6, IL-8, and IL-10, IL-15,IL-13, IL-17, IL-1Rα, MIP-1β, IL-2r,IL-7 Exotaxin, MCP-1, MIG, MIP-1alpha and RANTES, in a kit pre-tested for any potential cross-reactivity by the manufacturer. Controls included the standard curve and multiplex QC standards (R&D Systems).

## Abbreviations

PBMC: Peripheral blood mononuclear cells; AFP: Alpha fetoprotein; DC: Dendritic cells; Treg: Regulatory T cells; NK: Natural killer; HAdV-5: Human adenovirus serotype 5; MFI: Mean fluorescence intensity; HAdV-5 TMM2: Human adenovirus serotype 5/Tyrosinase, MART-1, MAGE-A6; HD: Healthy donors.

## Competing interests

The authors declare that they have no competing interests.

## Authors’ contributions

HPN performed the experiments, analyzed the data and wrote the manuscript; LV assisted with aspects of the study and with writing the manuscript. LH Butterfield conceived of the study, supervised its conduct, reviewed the data and wrote the manuscript. All authors read and approved the final manuscript.

## Authors’ information

Hadas Prag Naveh is a Dermatologist currently at Department of Dermatology, Rabin Medical Center, Beilinson Hospital, Israel. Lazar Vujanovic is a Research Instructor in Medicine, and Lisa H. Butterfield is a Professor of Medicine, Surgery, and Immunology at the University of Pittsburgh Cancer Institute (UPCI) and the University of Pittsburgh.

## Supplementary Material

Additional file 1: Figure S1 Anti-HAdV-5 neutralizing antibodies. Serum from each HD and patient was tested for the level of neutralizing antibodies to HAdV-5. Assay controls of no HAdV-5 (negative), no serum (positive) and pooled human AB serum are also shown. GFP expression in the A549 indicator cells is shown as MFI. The dilution of serum is on the X axis and the extent of HAdV-5eGFP transduction blockade is plotted. Click here for file

Additional file 2: Figure S2 NK cells activated and expanded *in vitro*. The CD56/CD16 gating strategy (of gated lymphocytes by forward and size scatter, not shown) is shown in A, and the number in each gate corresponds to the population analyzed in B and C. The cultured cells were assessed weekly, and the frequency of activated (CD69^+^) NK cells from each gate is shown in B, and the subset of those CD69^+^ cells expressing IFNγ is shown in C. Error bars represent standard errors. Click here for file
