# Supplementary material for: Cellular immunity induced by a recombinant adenovirus- human dendritic cell vaccine for melanoma
Source: J Immunother Cancer. 2013 Nov 18;1:19. doi: 10.1186/2051-1426-1-19 (PMC4019908; doi:10.1186/2051-1426-1-19)
Supplement: Additional file 1: Figure S1 — Anti-HAdV-5 neutralizing antibodies. Serum from each HD and patient was tested for the level of neutralizing antibodies to HAdV-5. Assay controls of no HAdV-5 (negative), no serum (positive) and pooled human AB serum are also shown. GFP expression in the A549 indicator cells is shown as MFI. The dilution of serum is on the X axis and the extent of HAdV-5eGFP transduction blockade is plotted. [file 2051-1426-1-19-S1.pptx]

## Slide 1
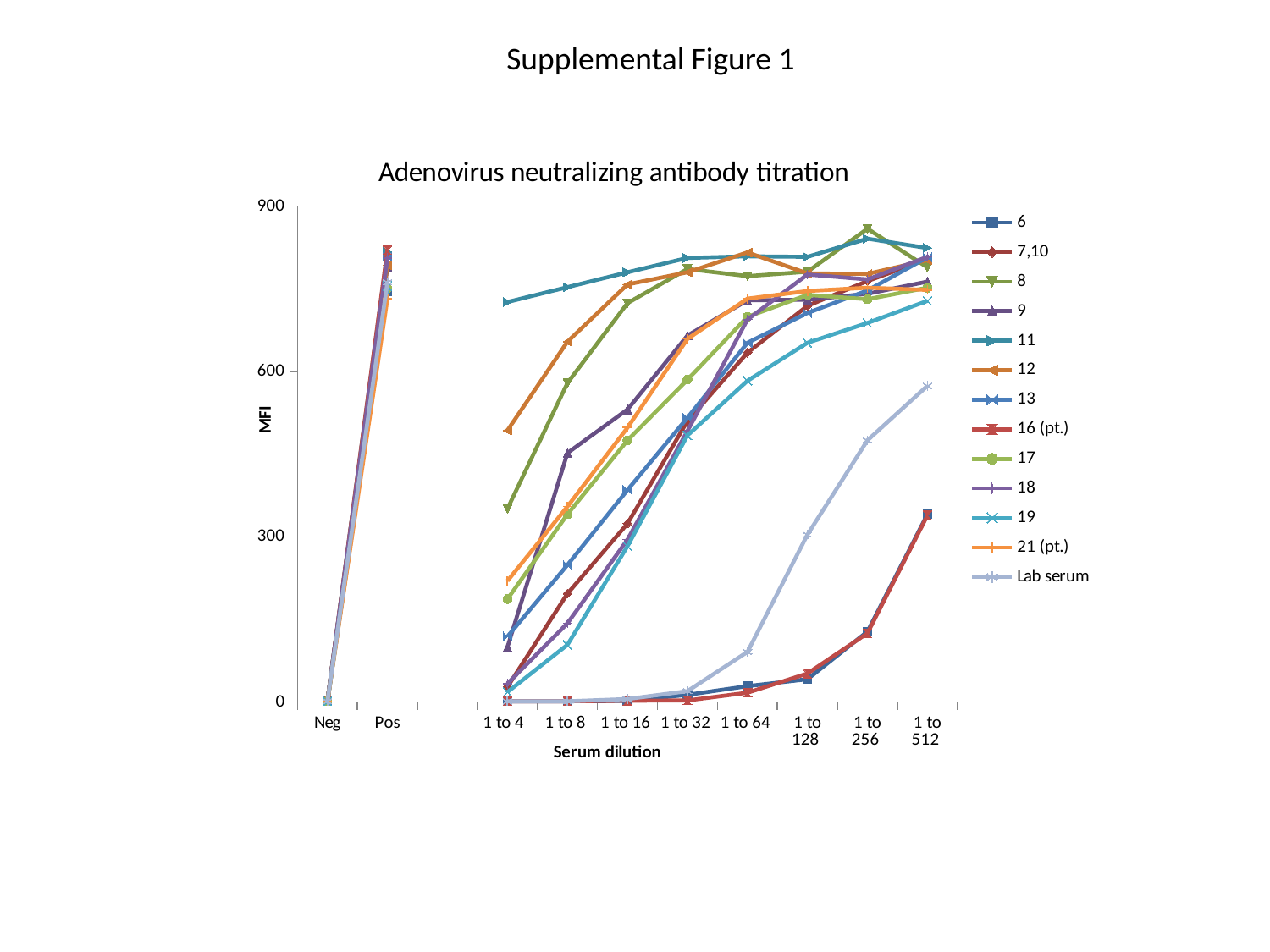

Supplemental Figure 1
### Chart: Adenovirus neutralizing antibody titration
| Category | 6 | 7,10 | 8 | 9 | 11 | 12 | 13 | 16 (pt.) | 17 | 18 | 19 | 21 (pt.) | Lab serum |
|---|---|---|---|---|---|---|---|---|---|---|---|---|---|
| Neg | 2.13 | 1.44 | 1.42 | 1.43 | 2.12 | 3.11 | 1.44 | 1.4 | 1.42 | 2.29 | 1.42 | 1.44 | 1.47 |
| Pos | 745.0 | 811.0 | 804.0 | 790.0 | 817.0 | 793.0 | 808.0 | 820.0 | 751.0 | 806.0 | 755.0 | 732.0 | 762.0 |
| | None | None | None | None | None | None | None | None | None | None | None | None | None |
| 1 to 4 | 1.44 | 27.6 | 351.0 | 99.82 | 726.0 | 493.0 | 119.0 | 1.45 | 187.0 | 33.4 | 18.3 | 220.0 | 1.4 |
| 1 to 8 | 1.48 | 197.0 | 579.0 | 452.0 | 753.0 | 654.0 | 249.0 | 1.47 | 341.0 | 143.0 | 104.0 | 355.0 | 1.53 |
| 1 to 16 | 2.2 | 324.0 | 724.0 | 531.0 | 780.0 | 758.0 | 385.0 | 2.58 | 475.0 | 295.0 | 283.0 | 498.0 | 5.59 |
| 1 to 32 | 13.2 | 510.0 | 786.0 | 665.0 | 806.0 | 780.0 | 516.0 | 3.03 | 585.0 | 492.0 | 484.0 | 659.0 | 19.9 |
| 1 to 64 | 29.0 | 634.0 | 773.0 | 729.0 | 809.0 | 816.0 | 652.0 | 17.1 | 699.0 | 694.0 | 583.0 | 732.0 | 91.1 |
| 1 to 128 | 41.6 | 719.0 | 781.0 | 730.0 | 808.0 | 778.0 | 706.0 | 51.9 | 739.0 | 776.0 | 652.0 | 746.0 | 304.0 |
| 1 to 256 | 128.0 | 764.0 | 859.0 | 741.0 | 841.0 | 777.0 | 747.0 | 125.0 | 731.0 | 767.0 | 688.0 | 752.0 | 475.0 |
| 1 to 512 | 341.0 | 804.0 | 789.0 | 763.0 | 824.0 | 802.0 | 807.0 | 339.0 | 752.0 | 808.0 | 728.0 | 748.0 | 574.0 |
