# Supplementary material for: Cellular immunity induced by a recombinant adenovirus- human dendritic cell vaccine for melanoma
Source: J Immunother Cancer. 2013 Nov 18;1:19. doi: 10.1186/2051-1426-1-19 (PMC4019908; doi:10.1186/2051-1426-1-19)
Supplement: Additional file 2: Figure S2 — NK cells activated and expanded in vitro. The CD56/CD16 gating strategy (of gated lymphocytes by forward and size scatter, not shown) is shown in A, and the number in each gate corresponds to the population analyzed in B and C. The cultured cells were assessed weekly, and the frequency of activated (CD69+) NK cells from each gate is shown in B, and the subset of those CD69+ cells expressing IFNγ is shown in C. Error bars represent standard errors. [file 2051-1426-1-19-S2.pptx]

## Slide 1
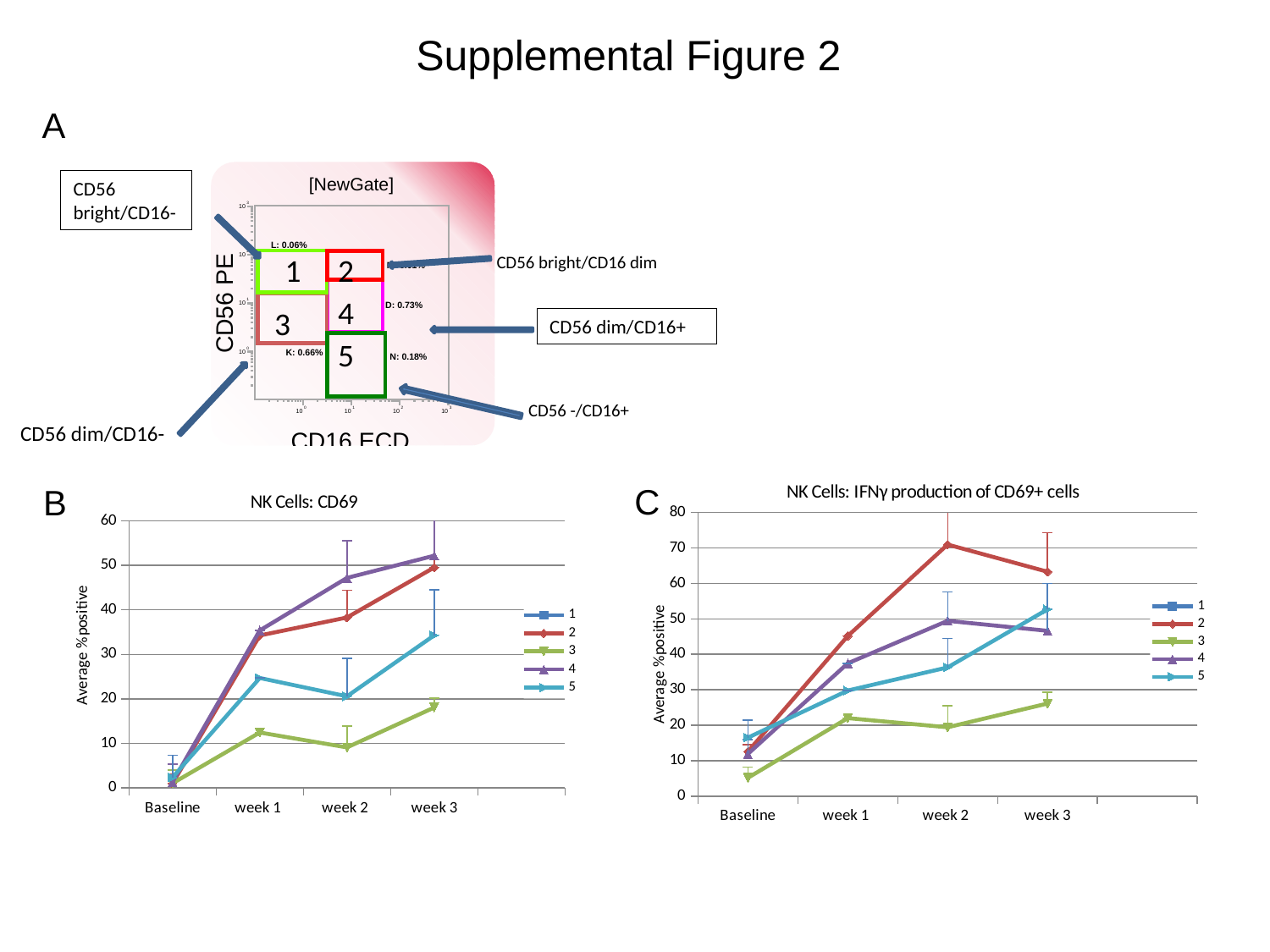

Supplemental Figure 2
A
CD56 bright/CD16-
CD56 bright/CD16 dim
CD56 dim/CD16+
CD56 -/CD16+
CD56 dim/CD16-
1
2
4
3
5
### Chart: NK Cells: IFNγ production of CD69+ cells
| Category | 1 | 2 | 3 | 4 | 5 |
|---|---|---|---|---|---|
| Baseline | 5.58 | 12.58 | 5.17 | 11.92 | 16.48999999999998 |
| week 1 | 35.99 | 45.19000000000001 | 22.0 | 37.46 | 29.79 |
| week 2 | 49.36 | 70.96 | 19.43 | 49.449999999999996 | 36.309999999999995 |
| week 3 | 56.839999999999996 | 63.3 | 26.12 | 46.64 | 52.69000000000001 |C
B
### Chart: NK Cells: CD69
| Category | 1 | 2 | 3 | 4 | 5 |
|---|---|---|---|---|---|
| Baseline | 0.7400000000000003 | 1.04 | 1.05 | 1.34 | 2.38 |
| week 1 | 13.51 | 34.25 | 12.450000000000005 | 35.379999999999995 | 24.69 |
| week 2 | 21.72 | 38.28 | 9.1 | 47.17 | 20.57 |
| week 3 | 42.29000000000001 | 49.5 | 18.06 | 52.15 | 34.27 |
